# The Institutional Library

**Published:** 1919-10-04

**Authors:** 


### THE INSTITUTIONAL LIBRARY.

**Housing and the Public Health.** By John Robertson, C.M.G., O.B.E., M.D. (Cassell and Co., Ltd. Price 5s. net.)

Housing is a question as multifarious as it is important, and when one finds a writer prepared to discuss it in a little book of 150 pages, one is rather reminded of the advertisement of Oxo—the ox in the cup. But Dr. Robertson has indeed put most of his ox into his cup; he has succeeded in boiling down his facts and concentrating much matter into a small volume. Scrappy though such a compendium must be, yet the essence of the matter is in the scraps, and the book is packed with useful information. It seems a pity, however, that the author did not contrive to squeeze in a more thorough discussion of the economic possibilities of careful and skilful planning and of ventilation. The housing question must always be mainly an economic one, and it seems to us that economic salvation is to be found chiefly in economic planning and scientific ventilation. Cubic capacity is not merely a question of the number of bricks employed, nor is ventilation merely a question of cubic capacity. Material can be economised by careful planning, and hygienic ventilation depends more on the rate of income of pure air than on the size of the room. Whether 6,000 cubic feet, or 600 cubic feet, or 60 cubic feet of air-space be sufficient for health depends on the rate of air exchange, and Mosso tells how forty-five persons were herded together in an Alpine hut which afforded them hardly one cubic metre of air each. We wish, for economic reasons, Dr. Robertson had dealt more fully with these aspects of the housing question. The second paragraph on page 34 is ambiguous and misleading. It can easily be read to convey exactly the opposite it seems meant to convey. We think, too, the author is incorrect when he says that the violet and ultra-violet light is stimulating to plant-life. The actinic rays cause heliotropism, but surely it is the red rays which specially stimulate plant growth. These, however, are details, and the book as a whole is accurate and is a valuable addition to the series of health manuals edited by Sir Malcolm Morris.

**Lectures on Venereal Disease.** By Leonard Myer, F.R.C.S. (London: The Albany Press. 1919. Pp. 88. Price 6s.)

Mr. Myer, it seems, has given a course of lectures on venereal disease, and doubtless with reluctance has yielded to the temptation to address a larger audience. His book is dedicated to the institution which provided him with the necessary platform, and the Chairman of the lay committee, in accepting this tribute, expresses the conviction that "the work will be of the utmost value." With great respect to the Chairman and his colleagues, we entirely disagree with this conclusion. That it is not of value to the medical profession the author himself recognises, though he would fain gather the "younger members" into his audience. It is "nurses, midwives, masseurs," and certain members of the "general public" that he is anxious to reach, and it is quite possible he may attain his aim. That such an audience has need of a detailed illustration of the male and female sexual organs, and of a scanty and superficial account of syphilis and gonorrhœa, and of their complications, we do not believe, and we judge the issue of such an account to "all types of readers" to be an ill-judged enterprise. Taking the book as it stands, and leaving aside the motives which have prompted it, there is little to be found beyond the ordinary information of the students' text-book, though certain commonplaces are announced with the portentous solemnity appropriate to an original discovery. The "lectures," in short, are mainly paste and scissors, with some personal experiences of the lecturer that may make a lay reader gasp but will hardly appeal to his sense of good taste. The Chairman, in commending the volume, speaks of its "value," and the publisher estimates this at 6s. Our own judgment inclines rather to a minus quantity.

**Present-Day Applications of Psychology:** With Special Reference to Industry, Education, and Nervous Breakdown. By Chas. S. Myers, M.A., M.D., Sc.D., F.R.S., Director of the Psychological Laboratory, Cambridge. (London: Methuen and Co., Ltd. Price 1s. net.)

This small booklet now appears in its third edition, and contains two lectures delivered at the Royal Institution of Great Britain on April 11 and April 18, 1918. At the outset the author traces the gradual evolution as an independent science of mental philosophy, or, as it is now called, psychology, and then proceeds to compare the standpoints at the present time of the physicists, the physiologist, and the psychologist. Particularly are these few paragraphs of interest in their purely medical bearing, although the extreme types of thinkers who are compared are not so commonly met with as the author's words might imply. However, his words are most valuable in drawing attention to the danger of too great concentration in one channel, and forms an apt introduction to the industrial considerations which follow. In view of the recent observations and suggestions which have abounded in regard to improved conditions among workpeople, this portion of the lectures (pp. 7-26) may be read with the greatest advantage by all who are directly or indirectly interested in the subject. The whole section is set down most clearly and excellently paragraphed and referenced, as it provides much valuable information, and proves over and over again that the future can only be in educating both employers and employees. The subject of nervous exhaustion, breakdown, and the indirect methods for their prevention are fully discussed on pages 29 and 47, and this portion of the lectures will probably hold the greater interest for the majority of the medical profession, and particularly the general practitioner, who so frequently encounters these conditions but has not always the opportunity adequately to study them.

**The Urethroscope, in the Diagnosis and Treatment of Urethritis.** By Major N. P. L. Lumb, O.B.E., R.A.M.C. (T.C.). (London: John Bale, Sons and Danielsson, Ltd. 1919. Price 10s. 6d.)

This small volume is most elegantly and readably produced and can, we feel, with its forty original illustrations in colour, justly claim to be a contribution to urethroscopy. Not that it brings any important new knowledge or methods to light, but, as claimed in the author's preface, in no work which has come to notice has an attempt been made to show the effect of treatment on the lesions of urethritis which can be observed with the urethroscope. It is commonly stated, for example, that dilatation should be controlled by regulated urethroscopic examination; yet there is little to guide the inexperienced surgeon as to what he may expect to see and when he may consider a lesion cured. With this situation in view the author, well assisted by his publishers, has set about providing a compact volume which shall cover the ground required. We may congratulate him on his success, and believe that

**The Institutional Library**—(*continued*).

if small sections of technical medical subjects were more often thus well and individually treated the result would be greatly to the practitioner's benefit.

**Elements of Pediatrics, for Medical Students.** By Rowland Godfrey Freeman, A.B., M.D. (New York: The MacMillan Company. Price 10s. 6d. net.)

This is a straightforward and simple study of the problem of keeping infants and children well by proper surroundings, *régime*, and feedings. A valuable feature is a summary of important facts to be obtained by physical examination, by examination of the urine and fæces, and by *x*-ray work. The book is obviously written for students, and as such may be strongly recommended, particularly for those about to take up the work, for its contents are mainly facts which should be made familiar to medical students before rather than after any special experience in the diseases of infancy and childhood has commenced.

**Shell-Shock.** By Professor G. Elliot Smith, M.D., F.R.S., and T. H. Pear, M.A., B.Sc. (London: Longmans, Green and Co. 1919. Pp. 135. Price 1s. 6d. net.)

This is a cheap reissue of an exposition of those cases of emotional disturbance due to the strain of modern war which the Army medical authorities most unfortunately allowed for the first two or three years of the war to be called "Shell-Shock." The failure of the British medical service to grasp the real nature of this condition, which was obvious from the first to many individual medical officers, resulted in a befogging of public opinion which has even yet not wholly dispersed. The original issue of this book did much to clarify the position; but even yet there are many pension problems arising out of war psychoses which still give rise to much popular misunderstanding. This reissue in a popular edition is therefore by no means out of place; for it is a trustworthy guide to much that has seemed to be unnecessarily mysterious and even terrifying.

**The People's Health.** By Walter Moore Coleman. New York: The Macmillan Company. Price 3s. 6d. net.)

This volume is No. 2 of the Practical Series, and been extensively used in the U.S.A schools since its publication in 1913. It is a one-book (half-year) course in personal, household, and industrial hygiene, public health, and human physiology. Particular care has been taken to reduce all the most recently established sanitary knowledge into a brief and clear code, and we think that its 360 odd pages have very great utility for school, and, in fact, all teaching purposes. This being the first aim of the author, perhaps no further comment or description is necessary here.

**Notes on Galvanism and Faradism.** By E. M. Maghill, M.B., B.S.Lond., D.P.H., R.S.C.I. (Second edition, with 67 illustrations. (London: H. K. Lewis and Co. 1919. Price 6s. net.)

This book is intended for the use of masseuses preparing for the examination of Medical Electricity now held by the Incorporated Society of Trained Masseuses, and the author deals with this difficult and complex subject in clear, concise language, free from technicalities. Electricity is discussed from its earliest inception as a therapeutic agent. There is also included a useful appendix dealing with electrical theories, the effect of the electric current on bacteria, and suggested electrical treatments where no precise prescription has been given; and also the questions set in the Medical Electricity examinations of the I.S.T.M. from 1915 to 1918. This is a book that will be found most useful to the qualifying student, who will be amply repaid by a study of its practical details.

**Infant and Young Child Welfare.** By Harold Scurfield, M.D. (London: Cassell and Co. 1919. Pp. 165. Price 5s. net.)

The keynote struck throughout this book, and struck with admirable emphasis, is the paramount duty of mothers to nurse their children at the breast, on account of the vastly improved prospects which maternal lactation ensures for the babies. There are, of course, many other topics touched upon concerning the welfare of infants; and they are dealt with adequately and carefully. On this topic of breast-feeding, however, Dr. Scurfield evidently feels very deeply indeed, doubtless because close at hand at Bradford (he is Medical Officer of Health for Sheffield) and other factory towns employing female labour in great quantity, the birth-rate and infantile death-rate conditions are a disgrace to a civilised nation. He shows that in poverty-stricken, dirty, ignorant Connaught the infantile death-rate is barely one-quarter of that in opulent, busy Bradford, where a model Infant Welfare Centre is kept up at a cost of £20,000 a year, and that the birth-rate is much higher in the Irish province: all because the Irish mother does not stifle her maternal instincts nor shirk her maternal duties. The book is helpful and practical throughout, and deserves a wide popularity.

**Idealus Odsfish, Author.** By Wilfred Beet Woollam, M.A., LL.M., Peterhouse, Cambridge. Price 7s. 6d. net. Author's address: North Road, Fairfield, Buxton, Derbyshire. (To be published by subscription.)

We have been invited by the author to read the opening chapters of this work, which form part of a circular containing a petition to the Prime Minister on his behalf for a literary petition, in consequence of "his age and circumstances," and in recognition of his "record of work." The petition, signed by many distinguished names, has, we understand, been successful. Of the first three chapters of his new book, it must suffice to say that they introduce a rambling narrative in a rambling style, both of which appear to have been influenced by Dickens and Sterne. Since Sterne sinned so grossly against all canons of order, canons of style, canons of sequence, that he became one of the immortals, it is idle to say that disorder, loose syntax, and inconsequence necessarily put a writer out of court. For in art there are no rules; there are only precedents; and there is an admitted precedent for the rambling style as for the careful. Sterne and Mathew Arnold are equally English classics. This said, readers must be guided by their preference. We merely inform them of the *genre* to which their attention is invited.

**Food.** By Alexander Hill, M.A., M.D., F.R.C.S. (London: Society for Promoting Christian Knowledge. 1917. Price 9d.)

"Food" is the title of the first number of a useful series of Health Manuals under the general editorship of Dr. Barclay-Smith, Professor of Anatomy in the University of London. In brief compass it conveys, with great simplicity of diction, sound information relating to the chemistry of food, nutrients, digestion, and various kinds of foodstuffs such as would be valuable to the new type of better educated cooks beginning to find their way into institutions. Housekeepers and caterers, no less than students, need more knowledge of food than their cook-books supply, and, within the space of sixty-four well-printed little pages, Dr. Alexander Hill has succeeded in bringing before his readers exactly what will be of use to them in a particularly interesting manner.

